# Allometric biomass partitioning under nitrogen enrichment: Evidence from manipulative experiments around the world

**DOI:** 10.1038/srep28918

**Published:** 2016-06-28

**Authors:** Yunfeng Peng, Yuanhe Yang

**Affiliations:** 1 State Key Laboratory of Vegetation and Environmental Change, Institute of Botany, Chinese Academy of Sciences, Beijing 100093, China

## Abstract

Allometric and optimal hypotheses have been widely used to explain biomass partitioning in response to resource changes for individual plants; however, little evidence has been reported from measurements at the community level across a broad geographic scale. This study assessed the nitrogen (N) effect on community-level root to shoot (R/S) ratios and biomass partitioning functions by synthesizing global manipulative experiments. Results showed that, in aggregate, N addition decreased the R/S ratios in various biomes. However, the scaling slopes of the allometric equations were not significantly altered by the N enrichment, possibly indicating that N-induced reduction of the R/S ratio is a consequence of allometric allocation as a function of increasing plant size rather than an optimal partitioning model. To further illustrate this point, we developed power function models to explore the relationships between aboveground and belowground biomass for various biomes; then, we generated the predicted root biomass from the observed shoot biomass and predicted R/S ratios. The comparison of predicted and observed N-induced changes of the R/S ratio revealed no significant differences between each other, supporting the allometric allocation hypothesis. These results suggest that allometry, rather than optimal allocation, explains the N-induced reduction in the R/S ratio across global biomes.

The partitioning of aboveground and belowground biomass is a central focus in plant ecology and evolution^1^,^2^. Not only does the partitioning pattern help to estimate the root biomass from the more easily measured shoot biomass but it also reflects the different investment of photosynthates between aboveground and belowground organs in response to changes in environmental conditions (*e.g.*, nitrogen (N) availability)^3^,^4^,^5^. Over the past few decades, N that entered the terrestrial environment has considerably increased^6^, and this trend is projected to rise to 102–156% by 2050 compared with the value for 2010 7. Hence, exploring how the external N input regulates aboveground and belowground biomass partitioning is of great importance for understanding plant-growth strategies and terrestrial carbon (C) cycling, particularly in light of global changes.

Aboveground and belowground biomass partitioning can be easily described in terms of the root to shoot (R/S) ratio^3^,^8^. Although individual studies indicated that N addition may decrease or have little influence on the R/S ratio in different plant species^3^,^5^,^9^, a meta-analysis revealed that N addition decreased it over a broad range^10^. However, whether the reduction of the R/S ratio is the consequence of optimal biomass partitioning between aboveground and belowground organs under N supplies or perhaps caused by a nonlinear (allometric) allocation as a function of increasing whole plant size remains contentious^3^,^4^,^11^. To solve this issue, researchers have established aboveground biomass (*M*_A_) and belowground (*M*_B_) allometric equations to test which hypothesis is more appropriate in explaining the N-induced changed in biomass partitioning patterns. Significant changes in the slope of allometric equations indicate an optimal biomass allocation under elevated N. Otherwise, changes in the R/S ratio result from allometric strategies under N addition^3^,^4^,^12^.

During the past few decades, abundant studies have been conducted to test both hypotheses in different plant species^2^,^3^,^5^,^13^. Nevertheless, the results from different experiments obtained inconsistent conclusions. For example, Shipley & Meziane^4^ found that N supply altered the scaling slopes of aboveground and belowground biomass allometric functions across 22 herbaceous species, which indicates an optimal C partitioning in response to N supplies. However, in Müller *et al*.^3^’s study, they observed that despite under N addition, there were reduced R/S ratios of all of the investigated plants, the allometric function was unaffected by changes in N availability in 21 out of the 27 species. These conflicting conclusions indicate that more studies are required from various perspectives. Given that most prior studies focused on biomass partitioning in response to N supplies from individual plants, it is still unknown how biomass allocation at the community level responds to external N addition. The community-level biomass partitioning may differ from individual plants because different species within a community have diverse life history strategies^14^,^15^. Recently, Zhou *et al*.^5^ found that, in the Gurbantunggut desert, N addition significantly altered the scaling slopes of aboveground and belowground allometric functions for annual species. Although it had little influence on ephemeral plants, the R/S ratios of different species were reduced by elevated N. Their results also demonstrated that, when pooling all species together, the N enrichment did not exert profound effects on the allometric equations, which possibly implies an allometric biomass allocation at the community level. However, no study has directly tested the two alternative hypotheses using systematic measurements at the community level. Particularly, it remains unclear whether allometric or optimal hypotheses could explain variations in community-level biomass partitioning patterns driven by N addition.

Here, we synthesized data from global N addition experiments to assess the influence of N enrichment on the R/S ratios and aboveground and belowground biomass allometric relationships. We also examined the N-induced changes in biomass partitioning patterns in relation to climatic and forcing variables. We aimed to test the following three hypotheses: (i) experimental N addition decreases the R/S ratio in global biomes; (ii) N addition has minor effects on the scaling slopes of the aboveground and belowground biomass allometric functions in various biomes; and (iii) N-induced reduction of the R/S ratio is primarily the result of allometric allocation as a function of increasing the whole plant size.

## Results

### Responses of the R/S ratios to N addition

At the global scale, external N addition significantly increased *M*_A_ for all biomes (*P* < 0.05). Likewise, *M*_B_ was evidently higher in forests and wetlands after N addition (*P* < 0.05); however, no pronounced differences were observed in grasslands (*P* = 0.38) and tundra (*P* = 0.27). N inputs significantly reduced the R/S ratio in grasslands and tundra (*P* < 0.05) and marginally decreased it in forests (*P* = 0.06); instead, N inputs had no pronounced impact on the R/S of wetlands (*P* = 0.13; Table 1).

### Responses of allometric equations to N addition

**Figure 1:**
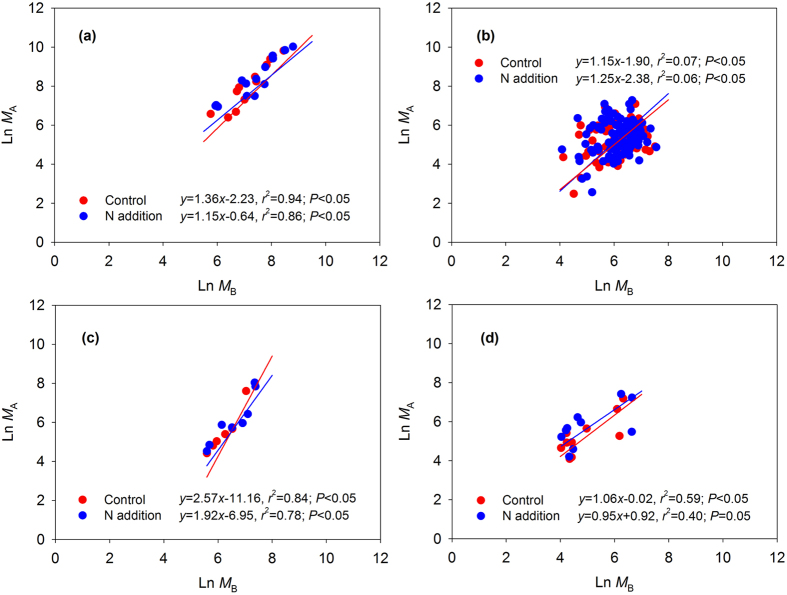
Reduced major axis (RMA) regression between aboveground biomass (*M*_A_) and belowground biomass (*M*_B_) for control and N addition treatments in forests (a), grasslands (b), wetlands (c) and tundra (d). Red and blue solid lines denote the regression curves of control and N addition treatments, respectively.

The scaling slopes of the *M*_A_-*M*_B_ allometric equations for forests, grasslands, wetlands and tundra were 1.36 (95% confidence interval (CI) of 1.18–1.56), 1.15 (95% CI of 0.95–1.39), 2.57 (95% CI of 1.73–3.81) and 1.06 (95% CI of 0.64–1.75) in ambient N treatment, respectively, and were 1.15 (95% CI of 0.92–1.45), 1.25 (95% CI of 1.04–1.52), 1.92 (95% CI of 1.21–3.03) and 0.95 (95% CI of 0.52–1.72) in elevated N treatment, respectively (Table 2, Fig. 1). However, the comparison of the scaling slopes of these functions did not show significant differences between the control and N addition for various biomes either by reduced major axis (RMA) analysis (Table 2) or analysis of covariance (Table 3).

### Comparison of observed and simulated N-induced changes of R/S ratios

**Figure 2:**
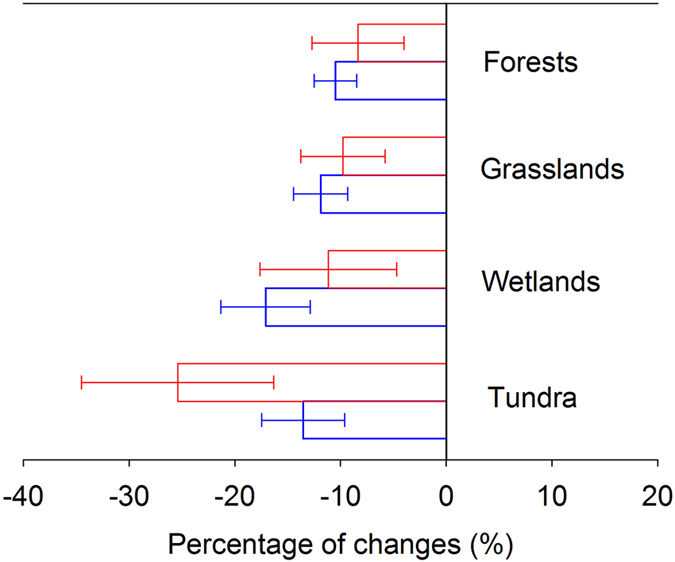
Observed and predicted N-induced changes in R/S ratio for forests, grasslands, wetlands and tundra. Red and blue open bars indicate observed and predicted values with standard errors, respectively. Paired samples T tests showed no significant differences between observed and predicted values for forests (*P* = 0.66), grasslands (*P* = 0.55), wetlands (*P* = 0.29) and tundra (*P* = 0.11).

By pooling the biomass data across control and N addition treatments, the relationships between *M*_A_ and *M*_B_ were characterized by the power functions (Forests: *M*_B_ = 3.5 × *M*_A_^0.73^, *r*^2^ = 0.89, *P* < 0.05; Grasslands: *M*_B_ = 152.5 × *M*_A_^0.21^, *r*^2^ = 0.07, *P* < 0.05; Wetlands: *M*_B_ = 36.3 × *M*_A_^0.48^, *r*^2^ = 0.87, *P* < 0.05; Tundra: *M*_B_ = 3.7 × *M*_A_^0.66^, *r*^2^ = 0.49, *P* < 0.05). Using these power functions, the predicted *M*_B_ was generated from *M*_A_. Afterwards, the R/S ratios for ambient and elevated N treatments were calculated. Compared with the observed values, no significant differences were detected in the percentage of N-induced changes in the predicted R/S ratios in forests (*P* = 0.66), grasslands (*P* = 0.55), wetlands (*P* = 0.29) and tundra (*P* = 0.11) (Fig. 2).

## Discussion

As expected, N addition significantly decreased the R/S ratio in grasslands and tundra, and marginally decreased the R/S ratio in forests. Meanwhile, the average of the R/S ratio in wetlands was also decreased, although no statistically significant difference was found between control and N addition (Table 1). However, the scaling slopes of the *M*_A_-*M*_B_ allometric functions were unaffected for all biomes (Tables 2 and 3). To test whether the reduction of the R/S ratio is a consequence of allometric allocation as a function of increasing plant biomass, the predicted and observed N-induced changes of the R/S ratios were compared, and we found that the predicted changes agreed with the observed values (Fig. 2). These results indicated that, in the global biomes of this analysis, the allometric theory preferably explained the R/S ratio changes under elevated N. Indeed, based on the absolute changes, *M*_B_ also increased with N addition (Table 1), which illustrates that although N addition could relieve a N limitation for major terrestrial biomes^16^,^17^, plants possibly still need to allocate similarly proportional C to belowground and aboveground biomass. An interesting question arises: why does C allocation between aboveground and belowground remain unchanged even in a sufficient N situation? Potential explanations include that in natural ecosystems, with the exception of N, other growth-limiting factors, such as water, and other mineral nutrient deficiencies could also occur after N addition. For example, natural grasslands are often thought to be co-limited by water and N^18^,^19^,^20^. The addition of N often increases leaf photosynthesis, which may induce greater demand for water in grasslands^21^, so more organic matter and energy would be invested in root systems for maintaining water uptake^22^,^23^. This outcome may result in higher belowground biomass allocation than in unfertilized grasslands. In contrast, it is usually assumed that N addition will accelerate phosphorous (P) limitation in tropical and subtropical forests^24^,^25^. Therefore, plants grown at these sites may need to allocate newly assimilated products to roots to maintain P absorption; thus, this situation causes an increase in belowground biomass partitioning under the condition of external N input. Overall, our results indicate that the allometric theory may be more appropriate than the optimal allocation hypothesis for explaining global biomass partitioning patterns under N enrichment.

The present analysis collected data from published studies, and all of these papers only reported the means without replications. However, certain studies with large replications have observed optimal biomass allocation under different nutrient availability^4^,^26^. Therefore, the question is whether the non-significant change in biomass allocation under N addition here is due to the lack of replication for each individual experiment. To address this issue, it is better to compare the allometric slopes across all repetitions between control and N addition treatments within each study and across all studies. Unfortunately, the experiments collected usually have few replications, probably due to the labour-consuming experiments, with ~5 replicates averaged across all of the collected studies (Fig. S1). Thus, even if we could obtain each repetition of the collected experiments, it will still not be possible to test the optimal hypothesis for every individual site due to data limitation. Actually, Müller *et al*.^3^ grew 27 herbaceous species under two nutrient levels, but they found that only 5 species exhibited optimal biomass allocation between root and leaf biomass, even though there were ~20 repetitions for each species and each nutrient level. We also extracted the data from their paper to compare the overall nutrient effect on the allocation slope of the pooled data of all species and repetitions, and the results showed no significant difference of the allometric slopes between the high and low nutrient treatments (*P* = 0.29, Fig. S2). This outcome may indicate that, even if there are enough replications, the optimal allocation will not appear for all situations. Thus, the non-significant change in biomass allocation under N addition in our study is possibly not due to the lack of replication for individual experiments, but mainly resulted from the diverse allometric patterns of individual species. This can also be illustrated by Zhou *et al*.^5^, who conducted a field experiment with 6 N addition levels that was replicated 10 times in a desert ecosystem, and they reported that, although different individual plants displayed diverse biomass partitioning patterns in response to N enrichment, the external N addition had no significant effect on the scaling slope of the pooled species data.

In summary, our results demonstrated that N addition generally reduced the R/S ratios for various biomes; however, it had a minor impact on allometric functions, which possibly reflects that the N-induced reduction of the R/S ratio resulted from the allometric allocation as a function of increasing the whole plant biomass. We further illustrated this point by developing simple power function models between *M*_A_ and *M*_B_ and generating the predicted *M*_B_ from the observed *M*_A_. Then, we predicted the R/S ratios. Interestingly, the predicted N-induced changes in the R/S ratio are in agreement with the observed values, which indicate that the allometric models interpreted the reduction of the R/S ratios by N addition in various biomes. Overall, the results obtained from the present analysis suggest that, at the community level, the reduction in the R/S ratio may be more parsimoniously explained as allometric strategies than optimal models under the circumstance of N addition.

## Materials and Methods

### Data collection

Peer-reviewed papers were exhaustively searched from ISI-Web of Science (Thomson Reuters, New York, NY, USA), Google Scholar (Google, Inc., Mountain View, CA, USA) and China Knowledge Resource Integrated database (Tsinghua Tongfang Knowledge Network Technology Co, Ltd., Beijing, China). To avoid bias in reference selection, the studies were collected based on the following criteria: (1) N was directly added in the experiments. Certain studies examined the interactions of N addition with other global change factors (*e.g.*, elevated CO_2_, climate warming, precipitation, etc.); only data from the control and N addition plots were included; (2) The data were presented synchronously on both above- and below-ground biomass at the community level, namely, the metric of biomass should be given as “g m^−2^”, “kg ha^−1^”, “Mg ha^−1^” or other measure that could be converted to “g m^−2^”. Biomass measured from individual plants only (*i.e.*, the unit is “g” or “g plant^−1^”) was excluded from this synthesis; (3) The aboveground biomass was measured by harvesting all plants within a certain area for grasslands, wetlands and tundra, and by allometric equations from stem volume for forests. The belowground biomass was obtained by collecting roots from soil cores or soil monoliths methods. Likewise, both coarse and fine roots should had been recorded in forests; (4) Studies performed at distinct sites or with different N rates or vegetation types were treated as independent^16^,^27^. When continuous measurements were conducted for different years in a study, data from the last year was used^10^,^28^.

**Figure 3:**
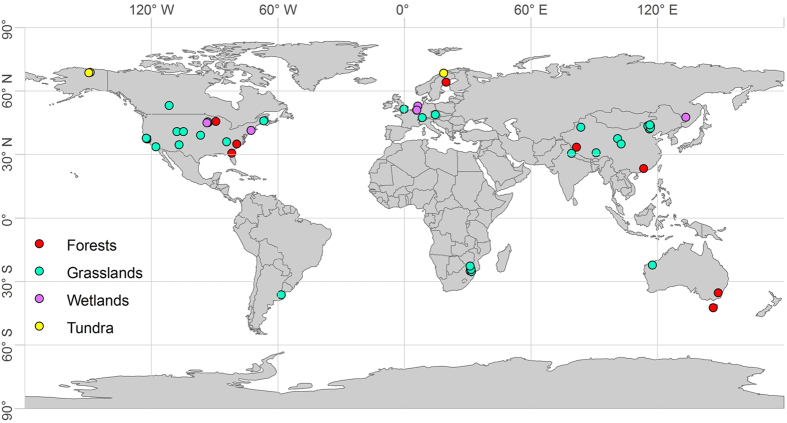
Site location of N addition experiments included in this study. The map was generated using ArcGIS 9.3 (http://resources.arcgis.com/en/home/).

In total, 136 pairwise observations from 56 studies were collected (Fig. 3; Supplementary Appendix S1–2). To test the potential differences of biomass partitioning among various biomes, four types of biomes, that is, forests, grasslands, wetlands and tundra, were differentiated in this analysis. Generally, most of the data originated from grasslands (103 pairwise observations), and less data originated from forests (15 pairwise observations), wetlands (8 pairwise observations) and tundra (10 pairwise observations). Due to limited data points in the forest biome, we did not divide them into subtypes (*e.g.*, boreal, temperate and tropical forests). The sites were located from 68.8°N to 42.5°S with MAT ranging from −11.5 to 25.3 °C and MAP from 160.5 to 1750.0 mm. For those studies that did not report climate characteristics, information was extracted from the global database at http://www.worldclim.org/ using longitude and latitude coordinates. All of the original data were converted to standard units (g m^−2^) before further analyses. In certain cases, the vegetation C pool size was reported, and we estimated dry biomass by the C pool divided by 0.45 29.

### Data analysis

Data were processed using the following three steps. First, to assess whether N enrichment significantly altered aboveground biomass, belowground biomass and the R/S ratio, we compared these variables between control and N addition treatments by paired samples T tests in forests, grasslands, wetlands and tundra. To meet the assumption of normal distribution, all the data were log_e_-transformed before the analyses.

Second, to examine whether N addition affected the scaling slopes of the allometric functions, we used two independent approaches to test the significance of the slopes difference between control and N treatment. First, we conducted an RMA analysis to examine the *M*_A_-*M*_B_ relationships for various biomes^30^,^31^,^32^. Generally, the allometric relationship between *M*_A_ and *M*_B_ can be described in the form of ln *y* = *a* + *b* (ln *x*), where *x* is *M*_B_, *y* is *M*_A_, *a* is the intercept, and *b* is the scaling slope^30^,^31^,^32^. Then, the comparison of the scaling slopes of the allometric functions was conducted using SMATR 2.0 33,34. In the process of RMA analysis, the biomass data were divided into two groups (control and N addition), and the *P* values for test of heterogeneity in slopes among groups were calculated from the likelihood ratio test and compared with a chi-squared distribution^33^. If *P* < 0.05, there was evidence that the group slopes were significantly different^35^. Second, to further investigate the proportionality of allocation and the influence of N addition on these relationships for various biomes, we fitted allometric regression models of the form of ln (*M*_A_) = *a* ln (*M*_B_) + *b* + *c* (N addition) + *e*, where *a* reflects the allometric slope, *b* represents the intercept, *c* represents the optimal partitioning response and *e* is the residual deviation^4^. Analysis of covariance (ANCOVA) was then used to examine the allometric slope and optimal partitioning response to the N treatment^3^,^36^. As in the RMA analysis, in ANCOVA, we divided the biomass data into control and N addition groups, and the ln (*M*_A_) was treated as the dependent variable, ln (*M*_B_) as the covariates and the group as fixed factor. If the interaction of group and ln (*M*_B_) was less than 0.05, it suggested that the slope was significantly different between control and N treatments and, thus, an optimal biomass allocation was considered under the N addition. Otherwise, N had no significant effect on the regression slopes^3^,^36^.

Third, to test whether the reduction of the R/S ratio under N enrichment was caused by allometric allocation with an increase in plant size, we performed ordinary least squares (OLS) regression to explore the relationship between *M*_A_ and *M*_B_ across ambient and elevated N treatments for forests, grasslands, wetlands and tundra. Consistent with previous studies^22^,^37^,^38^, a power function was used to fit the relationship between *M*_A_ and *M*_B_. We then calculated *M*_B_ from *M*_A_ by the above power functions and generated the predicted R/S ratios for ambient and elevated N treatments. We further calculated the percentage of N-induced changes of observed and predicted R/S ratios as (R/S_N_−R/S_C_)/R/S_C_ × 100 (R/S_N_ and R/S_C_ donated R/S ratios of control and N addition treatments, respectively). Then, the significance of the difference between the observed and predicted N-induced changes in the R/S ratio was compared by conducting a paired samples T test. We can deduce that the reduction in the R/S ratio under N addition mainly resulted from allometric allocation as a function of increasing the whole plant biomass if there are no significant differences between the predicted and observed N-induced changes of R/S ratios.

## Additional Information

**How to cite this article**: Peng, Y. and Yang, Y. Allometric biomass partitioning under nitrogen enrichment: Evidence from manipulative experiments around the world. *Sci. Rep.* **6**, 28918; doi: 10.1038/srep28918 (2016).

## Supplementary Material

Supplementary Information

## Figures and Tables

**Table 1:** Mean and 95% confidence level (CI) of aboveground biomass (*M*_A_, g m^−2^), belowground biomass (*M*_B_, g m^−2^) and R/S ratio under control and N addition treatments.

Biome	Treatment	*M*_A_		*M*_B_		R/S ratio		*n*
		Mean	95% CI	Mean	95% CI	Mean	95% CI	
Forests	Control	6044.7^b^	2391.9, 9697.6	1753.1^b^	928.6, 2577.7	0.45^a^	0.31, 0.59	15
	N addition	7788.0^a^	3600.2, 11976	2342.0^a^	1305.4, 3378.6	0.39^a^	0.28, 0.50	15
Grasslands	Control	229.8^b^	185.2, 274.5	559.9^a^	491.7, 628.1	3.83^a^	3.27, 4.39	103
	N addition	285.5^a^	235.3, 335.8	568.2^a^	501.4, 634.9	3.32^b^	2.77, 3.87	103
Wetlands	Control	646.7^b^	−58.3, 1351.6	644.0^b^	359.8, 928.1	2.09^a^	1.27, 2.91	8
	N addition	941.5^a^	−45.3, 1928.2	883.8^a^	432.3, 1335.3	1.80^a^	1.05, 2.54	8
Tundra	Control	330.7^b^	404.5, 620.9	205.9^a^	61.9, 350.0	0.85^a^	0.37, 1.32	10
	N addition	508.8^a^	111.8, 905.8	261.6^a^	49.0, 474.2	0.73^b^	0.08, 1.38	10

Different superscript lower-case letters denote significant differences in the means of the control and N-addition treatments (paired samples T test; *P* < 0.05).

**Table 2:** Reduced major axis (RMA) regression slopes (α_RMA_) and y-intercepts (log β_RMA_) of the relationships between aboveground biomass (*M*_A_) and belowground biomass (*M*_B_) under control and N addition treatments.

Biome	Control				N addition					*n*
	α_RMA_	95% CI	β_RMA_	95% CI	α_RMA_	95% CI	β_RMA_	95% CI	*P*_slope_	
Forests	1.36	1.18, 1.56	−2.23	−3.69, −0.77	1.15	0.92, 1.45	−0.64	−2.71, 1.43	0.20	15
Grasslands	1.15	0.95, 1.39	−1.90	−3.25, −0.55	1.25	1.04, 1.52	−2.38	−3.86, −0.90	0.51	103
Wetlands	2.57	1.73, 3.81	−11.16	−18.05, −4.27	1.92	1.21, 3.03	−6.95	−13.19, −0.71	0.27	8
Tundra	1.06	0.64, 1.75	−0.02	−2.82, 2.79	0.95	0.52, 1.72	0.92	−2.20, 4.04	0.76	10

The *P* values are shown for comparison of the scaling slopes between control and N addition by the likelihood ratio test.

**Table 3:** Allometric slope and optimal partitioning response examined with the analysis of covariance (ANCOVA) with ln (*M*_A_) as dependent variables, ln (*M*_B_) as covariates and treatments as fixed factors.

Biome	Source	MS	*F*	*P*
Forests	Allometric	36.1	212.1	<0.01
	Optimal	0.23	1.36	0.25
Grasslands	Allometric	9.1	14.0	<0.01
	Optimal	0.01	0.01	0.91
Wetlands	Allometric	19.0	87.7	<0.01
	Optimal	0.35	1.60	0.23
Tundra	Allometric	9.4	16.8	<0.01
	Optimal	0.22	0.39	0.54

*M*_A_, aboveground biomass; *M*_B_, belowground biomass.
