# Supplementary material for: Allometric biomass partitioning under nitrogen enrichment: Evidence from manipulative experiments around the world
Source: Sci Rep. 2016 Jun 28;6:28918. doi: 10.1038/srep28918 (PMC4923945; doi:10.1038/srep28918)
Supplement: Supplementary Information [file srep28918-s1.doc]

**Allometric biomass partitioning under nitrogen enrichment: Evidence from manipulative experiments around the world**

# Yunfeng Peng, and Yuanhe Yang


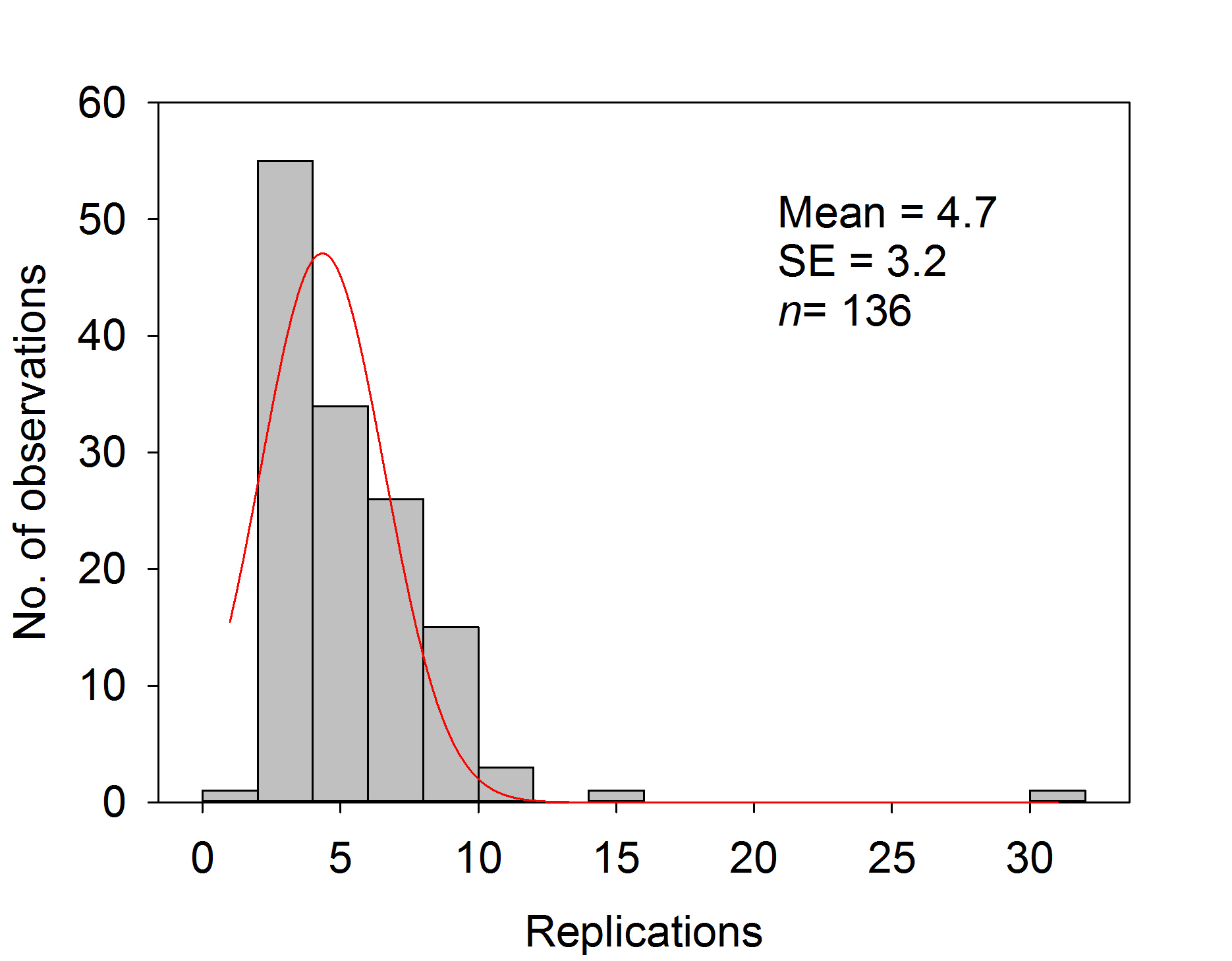


**Fig. S1** Frequency distribution of the replications for the collected experiments in our analysis.


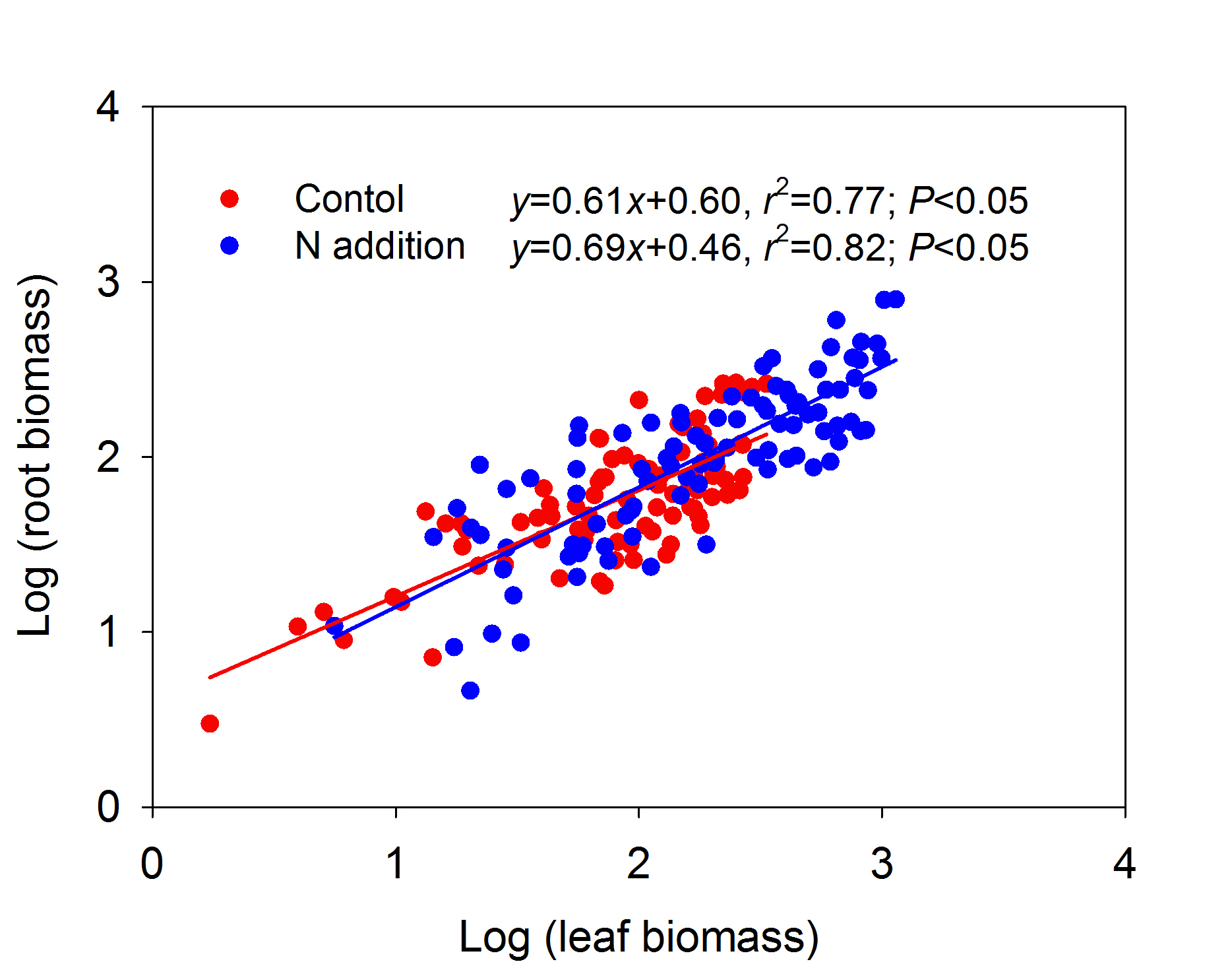


**Fig. S2** Relationships between Log-transformed root biomass and Log-transformed leaf biomass under control and N addition. Red and blue lines donate regression lines under control and N addition, respectively. Analysis of covariance (ANCOVA) showed non-significant difference between the regression slope of between control and N addition (*P*=0.29). Data are extracted from Müller *et al*. (2000).

**Appendix S1** Experimental conditions, climatic and forcing variables of papers from which the data were extracted for this study.

| **No.** | **Biome type** | **Site coordinate** | **MAT (^o^C)** | **MAP (mm)** | **Experimental duration (yr)** | **N rate (g m^-2^)** | **Cumulative N amount (g m^-2^ yr^-1^)** | **Note** | **References** |
| --- | --- | --- | --- | --- | --- | --- | --- | --- | --- |
| 1 | Forest | 23°20'N, 113°30'E | 21.5 | 1750 | 4 | 10 | 40 |  | Deng et al., 2010 |
| 2 | Forest | 30°34'N, 81°50'W | 19.6 | 1594 | 18 |  | 23.5 | Control | Shan et al., 2001 |
| 3 | Forest | 30°34'N, 81°50'W | 19.6 | 1594 | 18 |  | 23.5 | Understorey-eliminated | Shan et al., 2001 |
| 4 | Forest | 33º23'N, 81º40'E | 17.7 | 1303 | 3 | 8 | 24 | Vegetation type 1 | Coyle and Coleman, 2005 |
| 5 | Forest | 33º23'N, 81º40'E | 17.7 | 1303 | 3 | 8 | 24 | Vegetation type 2 | Coyle and Coleman, 2005 |
| 6 | Forest | 33º23'N, 81º40'E | 17.7 | 1303 | 3 | 8 | 24 | Vegetation type 3 | Coyle and Coleman, 2005 |
| 7 | Forest | 23º20'N, 113º30'E | 21.5 | 1750 |  |  |  |  | Liu et al, 2010 |
| 8 | Forest | 42°29'S,146°28'E | 10 | 1500 | 3 | 7.5 | 22.5 |  | Misra et al, 1998 |
| 9 | Forest | 64º14'N, 19º46'E | 1.3 | 590 | 15 | 1.25 | 18.75 | N level 1 | Gundale et al., 2013 |
| 10 | Forest | 64º14'N, 19º46'E | 1.3 | 590 | 15 | 5 | 75 | N level 2 | Gundale et al., 2013 |
| 11 | Forest | 45°38'N, 89°25'W | 4.5 | 790 | 2 | 5 | 10 | N level 1 | Coleman et al., 2004 |
| 12 | Forest | 45°38'N, 89°25'W | 4.5 | 790 | 2 | 10 | 20 | N level 2 | Coleman et al., 2004 |
| 13 | Forest | 45°38'N, 89°25'W | 4.5 | 790 | 2 | 20 | 40 | N level 3 | Coleman et al., 2004 |
| 14 | Forest | 34º54'N, 79º29'W | 16.2 | 1210 | 5 | 11.72 | 58.6 |  | Maier et al., 2004 |
| 15 | Forest | 35º21'S, 148º56'E | 9.6 | 791 | 11 | 30 | 330 |  | Ryan et al., 1996 |
| 16 | Grassland | 37°29'N, 101°12'E | -1.7 | 561 | 4 | 10 | 40 |  | Yang et al., 2014 |
| 17 | Grassland | 33°37'N, 117°45'W | 16.5 | 325 | 1 | 10 | 10 |  | Harpole et al., 2007 |
| 18 | Grassland | 43°26'N, 115°32'E | 0.7 | 343 | 5 | 2.5 | 12.5 | N level 1 | Chen et al., 2011 |
| 19 | Grassland | 43°26'N, 115°32'E | 0.7 | 343 | 5 | 5 | 25 | N level 2 | Chen et al., 2011 |
| 20 | Grassland | 43º38'N, 116º42'E | 2.1 | 375 | 11 | 5.6 | 61.6 | N level 1 | Wei et al., 2013 |
| 21 | Grassland | 43º38'N, 116º42'E | 2.1 | 375 | 11 | 11.2 | 123.2 | N level 2 | Wei et al., 2013 |
| 22 | Grassland | 43º38'N, 116º42'E | 2.1 | 375 | 11 | 22.4 | 246.4 | N level 3 | Wei et al., 2013 |
| 23 | Grassland | 43º38'N, 116º42'E | 2.1 | 375 | 11 | 39.2 | 431.2 | N level 4 | Wei et al., 2013 |
| 24 | Grassland | 43º38'N, 116º42'E | 2.1 | 375 | 11 | 56 | 616 | N level 5 | Wei et al., 2013 |
| 25 | Grassland | 45º24'N, 93º12'W | 6.3 | 749 | 28 | 1 | 28 | N level 1 | Fornara and Tilman, 2012 |
| 26 | Grassland | 45º24'N, 93º12'W | 6.3 | 749 | 28 | 2 | 56 | N level 2 | Fornara and Tilman, 2012 |
| 27 | Grassland | 45º24'N, 93º12'W | 6.3 | 749 | 28 | 3.4 | 95.2 | N level 3 | Fornara and Tilman, 2012 |
| 28 | Grassland | 45º24'N, 93º12'W | 6.3 | 749 | 28 | 5.4 | 151.2 | N level 4 | Fornara and Tilman, 2012 |
| 29 | Grassland | 45º24'N, 93º12'W | 6.3 | 749 | 28 | 9.5 | 266 | N level 5 | Fornara and Tilman, 2012 |
| 30 | Grassland | 45º24'N, 93º12'W | 6.3 | 749 | 28 | 17 | 476 | N level 6 | Fornara and Tilman, 2012 |
| 31 | Grassland | 45º24'N, 93º12'W | 6.3 | 749 | 28 | 27 | 756 | N level 7 | Fornara and Tilman, 2012 |
| 32 | Grassland | 43º33'N, 116º41'E | 2 | 350 | 1 | 5.25 | 5.25 | N level 1 | Pan et al., 2005 |
| 33 | Grassland | 43º33'N, 116º41'E | 2 | 350 | 1 | 10.5 | 10.5 | N level 2 | Pan et al., 2005 |
| 34 | Grassland | 43º33'N, 116º41'E | 2 | 350 | 1 | 17.5 | 17.5 | N level 3 | Pan et al., 2005 |
| 35 | Grassland | 43º33'N, 116º41'E | 2 | 350 | 1 | 28 | 28 | N level 4 | Pan et al., 2005 |
| 36 | Grassland | 22°17'S, 117°40'E | 25.3 | 350 | 2 | 5 | 10 |  | Bennett and Adams, 2001 |
| 37 | Grassland | 37°15'N, 121°45'W | 14.8 | 450 | 2 | 10 |  |  | Huenneke et al., 1990 |
| 38 | Grassland | 50°51'N, 5°54'E | 9.8 | 805 | 3 | 10 | 30 | Site 1 | Bobbink, 1991 |
| 39 | Grassland | 50°51'N, 5°54'E | 9.8 | 805 | 3 | 10 | 30 | Site 2 | Bobbink, 1991 |
| 40 | Grassland | 53°05'N, 111°33'W | 1.9 | 160.5 | 2 | 5.4 | 10.8 |  | Lamb et al., 2007 |
| 41 | Grassland | 42°02'N, 116°17'E | 2.1 | 385.5 | 2 | 5 | 10 | N level 1 | Fan et al., 2010 |
| 42 | Grassland | 42°02'N, 116°17'E | 2.1 | 385.5 | 2 | 15 | 30 | N level 2 | Fan et al., 2010 |
| 43 | Grassland | 43º33'N,116º40'E | 1.1 | 345 | 7 | 5.6 | 39.2 | N level 1 | He et al., 2013 |
| 44 | Grassland | 43º33'N,116º40'E | 1.1 | 345 | 7 | 11.2 | 78.4 | N level 2 | He et al., 2013 |
| 45 | Grassland | 43º33'N,116º40'E | 1.1 | 345 | 7 | 22.4 | 156.8 | N level 3 | He et al., 2013 |
| 46 | Grassland | 43º33'N,116º40'E | 1.1 | 345 | 7 | 39.2 | 274.4 | N level 4 | He et al., 2013 |
| 47 | Grassland | 43º33'N,116º40'E | 1.1 | 345 | 7 | 56 | 392 | N level 5 | He et al., 2013 |
| 48 | Grassland | 37º24'N, 122º14'W | 13.8 | 655 | 5 | 2 | 10 |  | Dukes et al., 2005 |
| 49 | Grassland | 43°26'N, 115°32'E | 2.1 | 375 | 1 | 10 | 10 | Site 1 | Li et al., 2011 |
| 50 | Grassland | 43°26'N, 115°32'E | 2.1 | 375 | 1 | 10 | 10 | Site 1 | Li et al., 2011 |
| 51 | Grassland | 34°20'N, 106°50'W | 13.2 | 250 |  | 5 |  |  | Ladwig et al., 2012 |
| 52 | Grassland | 45º55'N, 66º36'W | 5.4 | 1099 | 1 | 4 |  | N level 1 | Belanger and Richards, 2000 |
| 53 | Grassland | 45º55'N, 66º36'W | 5.4 | 1099 | 1 | 8 |  | N level 2 | Belanger and Richards, 2000 |
| 54 | Grassland | 36º16'S, 58º16'W | 15 | 900 | 1 | 25 | 25 |  | Semmartin et al., 2007 |
| 55 | Grassland | 37°40'N, 122°22'W | 19.3 | 677 | 6 | 7 | 42 |  | Henry et al., 2006 |
| 56 | Grassland | 42º53'N, 83º43'E | -4.8 | 265.7 | 2 | 1 | 2 | N level 1 | Gong et al., 2013 |
| 57 | Grassland | 42º53'N, 83º43'E | -4.8 | 265.7 | 2 | 3 | 6 | N level 2 | Gong et al., 2013 |
| 58 | Grassland | 42º53'N, 83º43'E | -4.8 | 265.7 | 2 | 9 | 18 | N level 3 | Gong et al., 2013 |
| 59 | Grassland | 42º53'N, 83º43'E | -4.8 | 265.7 | 2 | 15 | 30 | N level 4 | Gong et al., 2013 |
| 60 | Grassland | 34°55'N, 102°53'E | 2.4 | 531.6 | 1 | 5 | 5 | N level 1 | Xin et al., 2014 |
| 61 | Grassland | 34°55'N, 102°53'E | 2.4 | 531.6 | 1 | 10 | 10 | N level 2 | Xin et al., 2014 |
| 62 | Grassland | 34°55'N, 102°53'E | 2.4 | 531.6 | 1 | 15 | 15 | N level 3 | Xin et al., 2014 |
| 63 | Grassland | 39°05'N, 96°35'W | 12.1 | 863 |  | 5 |  |  | Bare and Blair, 2008 |
| 64 | Grassland | 35°58'N, 84°17'W | 17 | 1322 | 6 | 20 | 120 |  | Blue et al., 2011 |
| 65 | Grassland | 43°38'N, 116°42'E | 2.1 | 280 | 2 | 2.5 | 5 |  | Gao et al.,2011 |
| 66 | Grassland | 43°38'N, 116°42'E | 1.4 | 400 | 1 |  |  |  | Verburg et al., 2004 |
| 67 | Grassland | 25°07'S, 31°13'E | 21.1 | 737 | 3 | 10 | 30 | Site 1 | Craine et al., 2008 |
| 68 | Grassland | 23°45'S, 31°26'E | 21.1 | 737 | 3 | 10 | 30 | Site 2 | Craine et al., 2008 |
| 69 | Grassland | 25°18'S, 31°55'E | 21.1 | 737 | 3 | 10 | 30 | Site 3 | Craine et al., 2008 |
| 70 | Grassland | 24°24'S, 31°44'E | 21.1 | 737 | 3 | 10 | 30 | Site 4 | Craine et al., 2008 |
| 71 | Grassland | 22°47'S, 31°15'E | 21.1 | 737 | 3 | 10 | 30 | Site 5 | Craine et al., 2008 |
| 72 | Grassland | 40º49'N, 107º46'W | 12.5 | 309 | 4 | 10 | 40 |  | Vinton and Burke, 1995 |
| 73 | Grassland | 45°N, 93°W | 15.5 | 660 | 1 | 4 | 4 | Vegetation type 1 | Reich et al., 2000 |
| 74 | Grassland | 45°N, 93°W | 15.5 | 660 | 1 | 4 | 4 | Vegetation type 2 | Reich et al., 2000 |
| 75 | Grassland | 45°N, 93°W | 15.5 | 660 | 1 | 4 | 4 | Vegetation type 3 | Reich et al., 2000 |
| 76 | Grassland | 45°N, 93°W | 15.5 | 660 | 1 | 4 | 4 | Vegetation type 4 | Reich et al., 2000 |
| 77 | Grassland | 45°N, 93°W | 15.5 | 660 | 1 | 4 | 4 | Vegetation type 5 | Reich et al., 2000 |
| 78 | Grassland | 45°N, 93°W | 15.5 | 660 | 1 | 4 | 4 | Vegetation type 6 | Reich et al., 2000 |
| 79 | Grassland | 45°N, 93°W | 15.5 | 660 | 1 | 4 | 4 | Vegetation type 7 | Reich et al., 2000 |
| 80 | Grassland | 45°N, 93°W | 15.5 | 660 | 1 | 4 | 4 | Vegetation type 8 | Reich et al., 2000 |
| 81 | Grassland | 45°N, 93°W | 15.5 | 660 | 1 | 4 | 4 | Vegetation type 9 | Reich et al., 2000 |
| 82 | Grassland | 45°N, 93°W | 15.5 | 660 | 1 | 4 | 4 | Vegetation type 10 | Reich et al., 2000 |
| 83 | Grassland | 45°N, 93°W | 15.5 | 660 | 1 | 4 | 4 | Vegetation type 11 | Reich et al., 2000 |
| 84 | Grassland | 45°N, 93°W | 15.5 | 660 | 1 | 4 | 4 | Vegetation type 12 | Reich et al., 2000 |
| 85 | Grassland | 45°N, 93°W | 15.5 | 660 | 1 | 4 | 4 | Vegetation type 13 | Reich et al., 2000 |
| 86 | Grassland | 45°N, 93°W | 15.5 | 660 | 1 | 4 | 4 | Vegetation type 14 | Reich et al., 2000 |
| 87 | Grassland | 45°N, 93°W | 15.5 | 660 | 1 | 4 | 4 | Vegetation type 15 | Reich et al., 2000 |
| 88 | Grassland | 45°N, 93°W | 15.5 | 660 | 1 | 4 | 4 | Vegetation type 16 | Reich et al., 2000 |
| 89 | Grassland | 48º49'N, 14º39'E | 7.5 | 796 | 1 | 6.5 | 6.5 | Site 1 N level 1 | Picek et al., 2008 |
| 90 | Grassland | 48º49'N, 14º39'E | 7.5 | 796 | 1 | 30 | 30 | Site 1 N level 2 | Picek et al., 2008 |
| 91 | Grassland | 48º49'N, 14º39'E | 7.5 | 796 | 1 | 6.5 | 6.5 | Site 2 N level 1 | Picek et al., 2008 |
| 92 | Grassland | 48º49'N, 14º39'E | 7.5 | 796 | 1 | 30 | 30 | Site 2 N level 2 | Picek et al., 2008 |
| 93 | Grassland | 42º27'N, 116º41'E | 2.1 | 385 | 2 | 15 | 30 |  | Yan et al., 2010 |
| 94 | Grassland | 45º24'N, 93º12'W | 6.3 | 749 | 21 | 1 | 21 | N regime 1 N level 1 | Clark et al., 2009 |
| 95 | Grassland | 45º24'N, 93º12'W | 6.3 | 749 | 21 | 2 | 42 | N regime 1 N level 2 | Clark et al., 2009 |
| 96 | Grassland | 45º24'N, 93º12'W | 6.3 | 749 | 21 | 3.4 | 71.4 | N regime 1 N level 3 | Clark et al., 2009 |
| 97 | Grassland | 45º24'N, 93º12'W | 6.3 | 749 | 21 | 5.4 | 113.4 | N regime 1 N level 4 | Clark et al., 2009 |
| 98 | Grassland | 45º24'N, 93º12'W | 6.3 | 749 | 21 | 9.5 | 199.5 | N regime 1 N level 5 | Clark et al., 2009 |
| 99 | Grassland | 45º24'N, 93º12'W | 6.3 | 749 | 21 | 17 | 357 | N regime 1 N level 6 | Clark et al., 2009 |
| 100 | Grassland | 45º24'N, 93º12'W | 6.3 | 749 | 10 | 1 | 10 | N regime 2 N level 1 | Clark et al., 2009 |
| 101 | Grassland | 45º24'N, 93º12'W | 6.3 | 749 | 10 | 2 | 20 | N regime 2 N level 2 | Clark et al., 2009 |
| 102 | Grassland | 45º24'N, 93º12'W | 6.3 | 749 | 10 | 3.4 | 34 | N regime 2 N level 3 | Clark et al., 2009 |
| 103 | Grassland | 45º24'N, 93º12'W | 6.3 | 749 | 10 | 5.4 | 54 | N regime 2 N level 4 | Clark et al., 2009 |
| 104 | Grassland | 45º24'N, 93º12'W | 6.3 | 749 | 10 | 9.5 | 95 | N regime 2 N level 5 | Clark et al., 2009 |
| 105 | Grassland | 45º24'N, 93º12'W | 6.3 | 749 | 10 | 17 | 170 | N regime 2 N level 6 | Clark et al., 2009 |
| 106 | Grassland | 51º24'N, 0º12'W | 9.9 | 693 |  |  |  |  | Fornara et al., 2013 |
| 107 | Grassland | 30º46'N, 90º59'E | -0.6 | 414.6 | 3 | 1 | 3 |  | Wei et al., 2014 |
| 108 | Grassland | 47°22'N, 8°32'E | 9.4 | 1070 |  | 56 |  | N level 1 | Daepp et al., 2001 |
| 109 | Grassland | 47°22'N, 8°32'E | 9.4 | 1070 |  | 112 |  | N level 2 | Daepp et al., 2001 |
| 110 | Grassland | 43°26'N, 115°32'E | 0.7 | 335 |  | 7.5 |  | Grazing density 1 | Gong et al., 2015 |
| 111 | Grassland | 43°26'N, 115°32'E | 0.7 | 335 |  | 7.5 |  | Grazing density 2 | Gong et al., 2015 |
| 112 | Grassland | 43°26'N, 115°32'E | 0.7 | 335 |  | 7.5 |  | Grazing density 3 | Gong et al., 2015 |
| 113 | Grassland | 43°26'N, 115°32'E | 0.7 | 335 |  | 7.5 |  | Grazing density 4 | Gong et al., 2015 |
| 114 | Grassland | 30°28'N, 79°20'E | 13.1 | 1586 |  | 25 |  |  | Ram et al., 1991 |
| 115 | Grassland | 40º48'N, 104º42'W | 8.7 | 312 | 4 | 10 | 40 | Site 1 | Paschke et al., 2000 |
| 116 | Grassland | 40º48'N, 104º42'W | 8.7 | 312 | 4 | 10 | 40 | Site 2 | Paschke et al., 2000 |
| 117 | Grassland | 40º48'N, 104º42'W | 8.7 | 312 | 4 | 10 | 40 | Site 3 | Paschke et al., 2000 |
| 118 | Grassland | 40º48'N, 104º42'W | 8.7 | 312 | 4 | 10 | 40 | Site 4 | Paschke et al., 2000 |
| 119 | Wetland | 41°17'N, 72°41'W | 10.3 | 1169 | 5 |  | 82.5 |  | Anisfeld and Hill, 2012 |
| 120 | Wetland | 47°35'N, 133°31'E | 1.9 | 550 | 2 | 5 | 10 | N level 1 | Zhao et al., 2009 |
| 121 | Wetland | 47°35'N, 133°31'E | 1.9 | 550 | 2 | 15 | 30 | N level 2 | Zhao et al., 2009 |
| 122 | Wetland | 52º49'N, 6º25'E | 8.8 | 783 | 3 | 5 | 15 | Vegetation type 1 | Heijmans et al., 2002 |
| 123 | Wetland | 52º49'N, 6º25'E | 8.8 | 783 | 3 | 5 | 15 | Vegetation type 2 | Heijmans et al., 2002 |
| 124 | Wetland | 44°52'N, 93°36'W | 6.5 | 758 | 1 | 12 | 12 | N level 1 | Green and Galatowitsh, 2002 |
| 125 | Wetland | 44°52'N, 93°36'W | 6.5 | 758 | 1 | 48 | 48 | N level 2 | Green and Galatowitsh, 2002 |
| 126 | Wetland | 51°99'N, 5°70'E | 9 | 840 | 3 | 5 | 15 |  | Heijmans et al., 2001 |
| 127 | Tundra | 68°38'N, 149°34'W | -11.5 | 229 | 16 | 10 | 160 |  | Mack et al., 2004 |
| 128 | Tundra | 68°38'N, 149°34'W | -11.5 | 229 | 15 | 10 | 150 | Site 1 | Shaver et al., 1998 |
| 129 | Tundra | 68°38'N, 149°34'W | -11.5 | 229 | 15 | 10 | 150 | Site 2 | Shaver et al., 1998 |
| 130 | Tundra | 68°46'N, 148°52'W | -11.5 | 229 | 20 | 10 | 200 | Site 3 | Shaver et al., 1998 |
| 131 | Tundra | 68°38'N, 149°34'W | -11.5 | 229 | 8 | 10 | 80 | Site 1 | Nadelhoffer et al., 2002 |
| 132 | Tundra | 68°38'N, 149°34'W | -11.5 | 229 | 8 | 10 | 80 | Site 2 | Nadelhoffer et al., 2002 |
| 133 | Tundra | 68°38'N, 149°34'W | -4.4 | 229 | 8 | 10 | 80 | Site 3 | Nadelhoffer et al., 2002 |
| 134 | Tundra | 68°21'N, 18°40'E | -3 | 285 | 16 | 5 | 80 |  | Haugwitz et al, 2011 |
| 135 | Tundra | 68°38'N, 149°36'W | -11.5 | 229 | 5 | 10 | 50 |  | van Wijk et al., 2003 |
| 136 | Tundra | 68°38'N, 149°36'W | -11.5 | 229 | 4 |  |  |  | Chapin et al, 1986 |

**Appendix S2** A list of 56 papers from which the data were extracted for this study.

1. Anisfeld SC, Hill TD. 2012. Fertilization effects on elevation change and belowground carbon balance in a long Island sound tidal marsh. *Estuaries and Coasts* 35: 201–211.
2. Baer SG, Blair JM. 2008. Grassland establishment under varying resource availability: A test of positive and negative feedback. *Ecology* 89: 1859–1871.
3. Bélanger G, Richards JE. 2000. Dynamics of biomass and N accumulation of alfalfa under three N fertilization rates. *Plant and Soil* 219: 177–185.
4. Bennett LT, Adams MA. 2001. Response of a perennial grassland to nitrogen and phosphorus additions in sub-tropical, semi-arid Australia. *Journal of Arid Environments* 48: 289–308.
5. Blue JD, Souza L, Classen AT, Schweitzer JA, Sanders NJ. 2011. The variable effects of soil nitrogen availability and insect herbivory on aboveground and belowground plant biomass in an old-field ecosystem. *Oecologia* 167: 771–780.
6. Bobbink R. 1991. Effects of nutrient enrichment in dutch chalk grassland. *Journal of Applied Ecology* 28: 28–41.
7. Chapin III FS, Shaver GR, Kedrowski RA. 1986. Environmental controls over carbon, nitrogen and phosphorus fractions in *Eriophorum Vaginatum* in Alaskan tussock tundra. *Journal of Ecology* 74: 167-195.
8. Chen Q, Hooper DU, Lin S. 2011. Shifts in species composition constrain restoration of overgrazed grassland using nitrogen fertilization in Inner Mongolian steppe, China. *PLoS ONE* 6: e16909.
9. Clark CM, Hobbie SE, Venterea R, Tilman D. 2009. Long-lasting effects on nitrogen cycling 12 years after treatments cease despite minimal long-term nitrogen retention. *Global Change Biology* 15: 1755–1766.
10. Coleman MD, Friend AL, Kern CC. 2004. Carbon allocation and nitrogen acquisition in a developing *Populus deltoides* plantation. *Tree Physiology* 24: 1347–1357.
11. Coyle DR, Coleman MD. 2005. Forest production responses to irrigation and fertilization are not explained by shifts in allocation. *Forest Ecology and Management* 208: 137–152.
12. Craine JM, Morrow C, Stock WD. 2008. Nutrient concentration ratios and co-limitation in South African grasslands. *New Phytologist* 179: 829–836.
13. Daepp M, Nösberger J, Lüscher A. 2001. Nitrogen fertilization and developmental stage alter the response of *Lolium perenne* to elevated CO_2_. *New Phytologist* 150: 347–358.
14. Deng Q, Zhou G, Liu J, Liu S, Duan H, Zhang D. 2010. Responses of soil respiration to elevated carbon dioxide and nitrogen addition in young subtropical forest ecosystems in China. *Biogeosciences* 7: 315–328.
15. Dukes JS, Chiariello NR, Cleland EE, Moore LA, Shaw MR, Thayer S, Tobeck T, Mooney HA, Field CB. 2005. Responses of grassland production to single and multiple global environmental changes. *PLoS Biology* 10: e319
16. Fan W, Meng R, Chen QS. 2010. Effects of nitrogen additions on ground/underground biomass allocation of *stipa krylovii* community. *Animal Husbandry and Feed Science* 31: 74–75.
17. Fornara DA, Banin L, Crawley MJ. 2013. Multi-nutrient vs. nitrogen-only effects on carbon sequestration in grassland soils. *Global Change Biology* 19: 3848–3857.
18. Fornara DA, Tilman D. 2012. Soil carbon sequestration in prairie grasslands increased by chronic nitrogen addition. *Ecology* 93: 2030–2036.
19. Gao YZ, Chen Q, Lin S, Giese M, Brueck H. 2011. Resource manipulation effects on net primary production, biomass allocation and rain-use efficiency of two semiarid grassland sites in Inner Mongolia, China. *Oecologia* 165: 855–864.
20. Gong XY, Fanselow N, Dittert K, Taube F, Lin S. 2015. Response of primary production and biomass allocation to nitrogen and water supplementation along a grazing intensity gradient in semiarid grassland. *European Journal of Agronomy* 63: 27–35.
21. Gong YM, Mohammat A, Liu XJ, Li KH, Christie P, Fang F, Song W, Chang YH, Han WX, Lü XT, Liu YY, Hu KY. 2013. Response of carbon dioxide emissions to sheep grazing and nitrogen application in an alpine grassland. *Biogeosciences Discuss* 10: 12285–12311.
22. Green EK, Galatowitsch SM. 2002. Effects of *Phalaris arundinacea* and nitrate-N addition on the establishment of wetland plant communities. *Journal of Applied Ecology* 39: 134–144.
23. Gundale MJ, From F, Bach LH, Nordin A. 2013. Anthropogenic nitrogen deposition in boreal forests has a minor impact on the global carbon cycle. *Global Change Biology* 20: 276-286.
24. Harpole WS, Potts D, Suding KN. 2007. Ecosystem responses to water and nitrogen amendment in a California grassland. *Global Change Biology* 13: 2341–2348.
25. Haugwitz MS, Michelsen A, Schmidt IK. 2011. Long-term microbial control of nutrient availability and plant biomass in a subarctic-alpine heath after addition of carbon, fertilizer and fungicide. *Soil Biology & Biochemistry* 43: 179–187.
26. He N, Yu Q, Wang R, Zhang Y, Gao Y, Yu GR. 2013. Enhancement of carbon sequestration in soil in the temperature grasslands of northern china by addition of nitrogen and phosphorus. *PLoS ONE* 8: e77241.
27. Heijmans MMPD, Berendse F, Arp WJ, Masselink AK, Klees H, De Visser W, Van Breemen N. 2001. Effects of elevated carbon dioxide and increased nitrogen deposition on bog vegetation in the Netherlands. *Journal of Ecology* 89: 268–279.
28. Heijmans MMPD, Klees H, Berendse F. 2002. Competition between *Sphagnum magellanicum* and *Eriophorum angustifolium* as affected by raised CO_2_ and increased N deposition. *OIKOS* 97: 415–425.
29. Henry HAL, Chiariello NR, Vitousek PM, Mooney HA, Field CB. 2006. Interactive effects of fire, elevated carbon dioxide, nitrogen deposition, and precipitation on a California annual grassland. *Ecosystems* 9: 1066–1075.
30. Huenneke LF, Hamburg SP, Koide R, Mooney HA, Vitousek PM. 1990. Effects of soil resources on plant invasion and community structure in Californian serpentine grassland. *Ecology* 71: 478–491.
31. Ladwig LM, Collins SL, Swann AL, Xia Y, Allen MF, Allen EB. 2012. Above- and belowground responses to nitrogen addition in a Chihuahuan Desert grassland. *Oecologia* 169: 177–185.
32. Lamb EG, Shore BH, Cahill JF. 2007. Water and nitrogen addition differentially impact plant competition in a native rough fescue grassland. *Plant Ecology* 192: 21–33.
33. Li JZ, Lin S, Taube F, Pan QM, Dittert K. 2011. Above and belowground net primary productivity of grassland influenced by supplemental water and nitrogen in Inner Mongolia. *Plant and Soil* 340: 253–264.
34. Liu JL, Mei L, Gu JC, Quan XK, Wang ZQ. 2009. Effects of nitrogen fertilization on fine root biomass and morphology of *Fraxinus mandshurica* and *Larix gmelinii*: A study with in-growth core approach. *Chinese Journal of Ecology*, 2009, 28: 1–6.
35. Mack MC, Schuur EAG, Bret-Harte MS, Shaver GR, Chapin III FS. 2004. Ecosystem carbon storage in arctic tundra reduced by long-term nutrient fertilization. *Nature* 431: 440-443.
36. Maier CA, Albaugh TJ, Allen HL, Dougherty PM. 2004. Respiratory carbon use and carbon storage in mid-rotation loblolly pine (*Pinus taeda* L.) plantations: The effect of site resources on the stand carbon balance. *Global Change Biology* 10: 1335–1350.
37. Misra RK, Turnbull CRA, Cromer RN, Gibbons AK, LaSala AV. 1998. Below- and above-ground growth of *Eucalyptus Nitens* in a young plantation I. Biomass. *Forest Ecology and Management* 106: 283–293.
38. Nadelhoffer KJ, Johnson L, Laundre J, Giblin1 AE, Shaver GR. 2002. Fine root production and nutrient content in wet and moist arctic tundras as influenced by chronic fertilization. *Plant and Soil* 242: 107–113.
39. Pan QM, Bai YF, Han XG, Yang JC. 2005. Effects of nitrogen additions on a *Leymus Chinensis* population in a typical steppe of Inner Mongolia. *Acta Phytoecologica Sinica* 29: 311–317.
40. Paschke MW, McLendon T, Redente EF. 2000. Nitrogen availability and old-field succession in a shortgrass steppe. *Ecosystems* 3: 144–158.
41. Picek T, Kaštovská E, Edwards K, Zemanová K, Dušek J. 2008. Short term effects of experimental eutrophication on carbon and nitrogen cycling in two types of wet grassland. *Community Ecology* 9: 81-90.
42. Ram J, Singh SP, Singh JS. 1991. Effect of fertilizer on plant biomass distribution and net accumulation rate in an alpine meadow in central Himalaya, India. *Journal of Range Management* 44 140-143.
43. Reich PB, Tilman D, Craine J, Ellsworth D, Tjoelker MG, Knops J, Wedin D, Naeem S, Bahauddin D, Goth J, Bengtson W, Lee TD. 2001. Do species and functional groups differ in acquisition and use of C, N and water under varying atmospheric CO_2_ and N availability regimes? A field test with 16 grassland species. *New Phytologist* 150: 435–448.
44. Ryan MG, Hubbard RM, Pongracic S, Raison RJ, Mcmurtrie RE. 1996. Foliage, fine-root, woody-tissue and stand respiration in *Pinus radiata* in relation to nitrogen status. *Tree Physiology* 16: 333–343.
45. Semmartin M, Oyarzabal M, Loreti J, Oesterheld M. 2007. Controls of primary productivity and nutrient cycling in a temperate grassland with year-round production. *Austral Ecology* 32: 416–428.
46. Shan JP, Morris LA, Hendrick RL. 2001. The effects of management on soil and plant carbon sequestration in slash pine plantations. *Journal of Applied Ecology* 38: 932–941.
47. Shaver GR, Johnson LC, Cades DH, Murray G, Laundre JA, Rastetter EB, Nadelhoffer KJ, Giblin AE. 1998. Biomass and CO_2_ flux in wet sedge tundras: Responses to nutrients, temperature, and light. *Ecological Monographs* 68: 75-97.
48. van Wijk MT, Williams M, Gough L, Hobbie SE, Shaver GR. 2003. Luxury consumption of soil nutrients: A possible competitive strategy in above-ground and below-ground biomass allocation and root morphology for slow-growing arctic vegetation? *Journal of Ecology* 91: 664-676.
49. Verburg PJ, Arnone III JA, Obrist D, Schorran DE, Evans RD, Leroux-Swarthoutw D, Johnson DW, Luo YQ, Coleman J. 2004. Net ecosystem carbon exchange in two experimental grassland ecosystems. *Global Change Biology* 10: 498–508.
50. Vinton MA, Burke IC. 1995. Interactions between individual plant species and soil nutrient status in shortgrass steppe. *Ecology* 76: 1116-1133.
51. Wei CZ, Yu Q, Bai E, Lu XT, Li Q, Xia JY, Kardol P, Liang WJ, Wang ZW, Han XG. 2013. Nitrogen deposition weakens plant–microbe interactions in grassland ecosystems. *Global Change Biology* 19: 3688–3697.
52. Wei D, Xu-Ri, Liu YW, Wang YH, Wang YS. 2014. Three-year study of CO_2_ efflux and CH_4_/N_2_O fluxes at an alpine steppe site on the central Tibetan Plateau and their responses to simulated N deposition. *Geoderma* 232–234: 88–96.
53. Xin XJ, Wang G, Yang YB, Ren ZW. 2014. Effects of N, P addition on above/below-ground biomass allocation in a sub- alpine meadow. *Ecological Science* 33: 452–458.
54. Yan LM, Chen SP, Huang JH, Lin GH. 2010. Differential responses of auto- and heterotrophic soil respiration to water and nitrogen addition in a semiarid temperate steppe. *Global Change Biology* 16: 2345–2357.
55. Yang XX, Ren F, Zhou HK, He JS. 2014. Responses of plant community biomass to nitrogen and phosphorus additions in an alpine meadow on the Qinghai-Xizang Plateau. *Chinese Journal of Plant Ecology* 38: 159–166.
56. Zhao GY, Liu JH, Wang Y, Dou JX, Dong XY. 2009. Effects of elevated CO_2_ concentration and nitrogen supply on biomass and active carbon of freshwater marsh after two growing seasons in Sanjiang Plain, Northeast China. *Journal of Environmental Sciences* 21: 1393–1399.
